# Restrictions on antimicrobial use in food animal production: an international regulatory and economic survey

**DOI:** 10.1186/1744-8603-9-48

**Published:** 2013-10-16

**Authors:** Dina Fine Maron, Tyler JS Smith, Keeve E Nachman

**Affiliations:** 1 Johns Hopkins Center for a Livable Future, Johns Hopkins University, 615 North Wolfe Street, Suite W7010, Baltimore, MD 21205, USA; 2 Department of Environmental Health Sciences, Bloomberg School of Public Health, Johns Hopkins University, 615 North Wolfe Street, Baltimore, MD 21205, USA; 3 Department of Health Policy and Management, Bloomberg School of Public Health, Johns Hopkins University, 624 North Broadway, Baltimore, MD 21205, USA

**Keywords:** Antimicrobial, Antibiotic, Drug resistance, Bacteria, Food animal, Agriculture, Veterinarian, Trade

## Abstract

**Background:**

The administration of antimicrobial drugs to food animals at low doses for extended durations for growth promotion and disease prevention has been linked to the global health crisis of antimicrobial resistance. Internationally, multiple jurisdictions have responded by restricting antimicrobial use for these purposes, and by requiring a veterinary prescription to use these drugs in food animals. Opponents of these policies have argued that restrictions have been detrimental to food animal production where they have been adopted.

**Methods:**

We surveyed the antimicrobial use policies of 17 political jurisdictions outside of the United States with respect to growth promotion, disease prevention, and veterinary oversight, and reviewed the available evidence regarding their production impacts, including measures of animal health. Jurisdictions were included if they were a top-five importer of a major U.S. food animal product in 2011, as differences between the policies of the U.S. and other jurisdictions may lead to trade barriers to U.S. food animal product exports. Jurisdictions were also included if information on their policies was publicly available in English. We searched the peer-reviewed and grey literatures and corresponded with jurisdictions’ U.S. embassies, regulators, and local experts.

**Results:**

Jurisdictions were categorized by whether they prohibit use of antimicrobials for growth promotion and/or use of antimicrobials without a veterinary prescription. Of the 17 jurisdictions surveyed, six jurisdictions have prohibited both types of use, five jurisdictions have prohibited one use but not the other use, and five jurisdictions have not prohibited either use, while information was not available for one jurisdiction. Data on the production impacts of these prohibitions were limited, although available data, especially from Denmark and Sweden, suggest that restrictions on growth promotion use can be implemented with minimal production consequences.

**Conclusions:**

A majority of leading U.S. trade partners have more stringent policies regarding antibiotic use and veterinary oversight in food animal production. Available data suggest that restrictions on growth promotion may not be detrimental to production in the long run, although additional research could be useful. There is evidence that discordance between the U.S. and other jurisdictions with respect to antimicrobial use in food animals may be detrimental to U.S. access to export markets for food animal products. The available economic evidence strengthens the rationale for restricting antimicrobial use in U.S. food animals.

## Background

The dominant model of food animal production in the United States and increasingly in other countries is characterized by large-scale, high-throughput confinement operations with herds or flocks that can range in size from hundreds to hundreds of thousands of animals [1,2]. These animals are typically supplied with drinking water and grain-based feeds that may be amended with antimicrobial and other drugs for multiple purposes [3]. In the U.S., antimicrobials are approved to treat, control, and prevent disease in food animals, and for production purposes like growth promotion [4]. Many antimicrobials are available without a veterinary prescription [4]. In the U.S., the quantity of antimicrobials sold for use in food animals is approximately four times greater than the quantity sold for use in humans [5,6].

The widespread use of antimicrobials in food animal production has been linked to the development of antimicrobial resistance (AMR) in bacterial populations; AMR has emerged as a global health crisis [7]. When antimicrobials are administered to food animals for disease prevention or growth promotion, they are commonly administered at lower doses and for longer durations than when these drugs are used for disease treatment and control; administration of low doses for extended periods can increase selective pressure for AMR [8,9]. In these cases, antimicrobials are usually administered via medicated feed or drinking water on a herd- or flock-wide basis, leading to imprecise dosing when animals can choose what quantity of feed or water to consume and potentially enhancing selection for AMR [10]. Additionally, although veterinary oversight of antimicrobial use has been associated with reduced selection for AMR [11,12], veterinary involvement in the use of antimicrobials by the U.S. food animal industry is often limited. For example, a U.S. Department of Agriculture investigation found that only 46% of surveyed dairy producers based antibiotic use for a common condition in dairy cows on veterinary recommendations [13].

There is evidence that modifying some current production practices can limit food animal morbidity and mortality despite reductions in antimicrobial use [14,15]. Multiple countries have restricted the use of antimicrobials for growth promotion and disease prevention and/or required producers to obtain a veterinary prescription to purchase or administer these drugs. In the U.S., public health scientists and advocates have argued that, to protect the efficacy of antimicrobials at treating human diseases, all growth promotion uses and most prophylactic uses should be phased out of U.S. food animal production in favor of reforming production practices, and that veterinary oversight of all antimicrobial use should be required [16,17]. The U.S. Food and Drug Administration (FDA) has initiated a process by which pharmaceutical companies may voluntarily withdraw approvals to market antimicrobials for growth promotion and amend approvals to market antimicrobials over the counter so that a veterinary prescription or similar oversight is required for their purchase or use. The FDA has resisted calls to use its regulatory authority to withdraw these approvals, however, and has endorsed the continued use of antimicrobials for disease prevention [4]. This approach has drawn criticism from public health advocates, who promote countries with more restrictive policies as models for the U.S. to emulate [18,19].

The U.S. food animal and pharmaceutical industries have opposed restrictions adopted outside of the U.S. and have argued that such restrictions have been detrimental to food animal production where they have been implemented [20,21]. The production impacts of these policies have been discussed elsewhere to a limited extent [22], but we are unaware of any comprehensive review of these restrictions or their impacts on production in the peer-reviewed literature. This article surveys the policies of political jurisdictions regarding the use of antimicrobial drugs in food animal production. The available data on how these policies have affected animal health and the economic performance of these jurisdictions’ food animal industries are summarized by country.

## Methods

### Selection of political jurisdictions

In the U.S., antimicrobials are approved for use in the production of commodities including: poultry, eggs, pork, beef, and dairy products [23]. The fresh, chilled, and frozen forms of these commodities were included in this analysis, representing approximately 70% of U.S. terrestrial food animal product exports in 2011 [24]. Commodity data were obtained through the U.S. Department of Agriculture’s Foreign Agricultural Service’s (FAS) standard query search engine [24], which derives data from the U.S. Census Bureau’s Foreign Trade Statistics. This FAS data, characterized by the standard coding used to classify international trade—the Harmonized Commodity Description and Coding System (HS)—was the best fit for this work due to its international consistency, the accessibility of recent data, and the ease of communicating with FAS experts in the U.S. As defined here, “poultry” includes “poultry and products,” encompassing broiler meat, turkey meat, other poultry meat, live poultry, and miscellaneous poultry. “Pork” includes frozen, chilled, and fresh pork. “Beef” includes beef and/or veal that may be fresh, chilled, or frozen. “Eggs” includes eggs and egg products. “Dairy” includes milk and dairy-related products, including: condensed and evaporated milk, non-fat dry milk, dry whole milk and cream, fluid milk and cream, yogurt and other fermented milk, butter and milkfat, ice cream, cheese and curd, casein, whey, and other dairy products.

In recent years, multiple practices of the U.S. food animal industry, such as the use of exogenous hormones in cattle and the use of antimicrobial rinses to clean poultry carcasses, have led other countries to restrict the import of U.S. food animal products [25,26]. The possibility of similar barriers to U.S. products due to differences in antimicrobial use policies between the U.S. and other jurisdictions has been raised [22]. We selected jurisdictions based on their share of U.S. food animal product exports in part to help evaluate the possibility of potential future restrictions on these exports due to antimicrobial use.

We included jurisdictions listed by the U.S. Department of Commerce as among the top five importers of U.S. food animal products for at least one of the five included commodities in 2011 (Table 1). Jurisdictions were also included if information on their antimicrobial use policies was publicly available or we were able to obtain relevant information through contact with food animal production experts or government agencies of those countries. The EU was included as an independent entity although some of its 27 member states may have tighter antimicrobial restrictions or exemptions from EU policies. Several EU member states with relevant policies are included separately.

**Table 1:** The top 5 markets in 2011 for U.S. food animal exports

**Commodity**	**Jurisdiction**	**Sales (in $1,000s)**	**% of U.S. export sales for commodity**
**Dairy**	Mexico	1,165,916	24.4
	Canada	444,149	9.3
	China	361,993	7.6
	Philippines	280,306	5.9
	Japan	277,147	5.8
**Poultry**	Mexico	912,570	16.2
	Hong Kong	822,405	14.6
	Canada	581,601	10.3
	Russia	263,458	4.7
	China	249,192	4.4
**Pork**	Japan	1,884,048	40.2
	Mexico	599,267	12.8
	Canada	467,193	10.0
	China	464,389	9.9
	South Korea	433,277	9.2
**Beef**	Canada	865,985	19.0
	Mexico	760,606	16.7
	Japan	758,696	16.6
	South Korea	648,819	14.2
	EU	227,394	5.0
**Eggs**	Canada	74,691	17.2
	Japan	72,462	16.6
	EU	68,552	15.7
	Mexico	42,310	9.7
	Hong Kong	35,388	8.1

### Data collection on jurisdiction-specific policies

Available information on the included jurisdictions was collected via full-text searches of PubMed using the search terms “antimicrobial” and “livestock,” and/or “food production” and/or “growth promotion,” alongside the names of jurisdictions and commodities (poultry, chicken, turkey, beef, pork, swine, pig, dairy, and eggs). We identified additional articles by searching each article’s reference section. We also searched the grey literature and examined jurisdictions’ regulatory websites for relevant information. Additional information was obtained through direct contact with food animal production policy experts identified through their authorship of papers on this topic or through recommendations from other experts. We initially emailed or called embassies and/or jurisdiction-level experts and asked them about their respective national policies related to antimicrobial use for growth promotion and veterinary prescription requirements. We later inquired about policies related to antimicrobial use for disease prevention and control, specifically.

We examined jurisdiction-specific policies that impact the frequency of antimicrobial use for 1) production purposes, such as growth promotion or improved feed conversion, or 2) preventive reasons in the absence of veterinarian-diagnosed disease. It is important to recognize that the terminology used to characterize antimicrobial use in policy settings is divisive, contentious, and applied inconsistently. Given this, we endeavored to gather data from jurisdictions that could best describe their policies regarding these two contexts. In doing so, we used the specific terminology in policy documents or communications with jurisdiction-level experts.

Two frequent inconsistencies in terminology require clarification. The term *antimicrobial* refers to substances that are capable of inhibiting or killing microorganisms. *Antibiotics* are a subset of antimicrobials, and have traditionally included only antimicrobials that were originally derived from microorganisms. In our paper, we define the term *disease prevention* as the prophylactic administration of antimicrobials in the absence of disease. We use the term *disease control* to refer to administration of antimicrobials to a group of animals to control the spread of a disease when one or more individuals within a flock or herd have been diagnosed with disease.

The presentation of information regarding jurisdiction-specific policies varies in degree of detail because 1) uneven levels of information were available regarding the intricacies of national policies and 2) there exists an inconsistent degree of specificity in the actual policies. In some instances we were not given access to a copy of the regulations themselves, we were unable to access them in English, or we were forced to rely upon potentially incomplete information. In this work we report on the available national policies as they were reported to us. Provincial policies were not included in our analysis.

The policies of the 17 political jurisdictions in this work are classified by their antimicrobial restriction level in this paper, organized into three tiers and then listed in descending order by their contribution to overall U.S. export market sales across the five commodities (Table 2). Jurisdictions included in the Antimicrobial Use Restricted category have banned the use of antimicrobials for growth promotion and require veterinary prescriptions to use antimicrobials in food animals. The Semi-Restricted category includes jurisdictions that have a ban or partial ban on antimicrobial growth promoters (AGPs) or require veterinary prescriptions. The No Current Antimicrobial Use Restrictions category refers to jurisdictions that have no AGP restrictions or veterinary prescription requirements. For each jurisdiction, the analysis provides an overview of its antimicrobial use policies (including use-specific restrictions and veterinary prescription policies), the implementation timeline of these policies, and information on their production impacts, as available.

**Table 2:** Antimicrobial use in food animals and U.S. export sales for 2011

**Jurisdiction ranked by U.S. export market sales ($)**	**National ban on growth promotion**	**National veterinary prescription requirement**	**U.S. export sales for 5 commodities in $1,000s (% U.S. export market)**	**U.S. export sales for poultry incl. eggs in $1,000s (metric tons)**	**U.S. export sales for dairy in $1,000s (metric tons)**	**U.S. export sales for pork in $1,000s (metric tons)**	**U.S. export sales for beef in $1,000s (metric tons)**	**U.S. export sales for eggs in $1,000s (quantity in metric tons excl. eggs measured in dozens)**^**a**^
Mexico	Yes*	Yes	3,438,395 (17.1)	912,570 (657,711)	1,165,916 (370,602)	599,267 (286,197)	760,606 (156,029)	42,310 (982)
Japan	No	Yes	3,071,017 (15.2)	151,126 (84,270)	277,147 (107,417)	1,884,048 (464,593)	758,696 (138,910)	72,462 (17,333)
Canada	No	No	2,358,928 (11.7)	581,601 (180,929)	444,149 (137,927)	467,193 (120,914)	865,985 (145,540)	74,691 (4,744)
South Korea	N/D	N/D	1,455,922 (7.2)	151,497 (112,965)	222,329 (68,634)	433,277 (148,577)	648,819 (135,553)	5,175 (1,794)
Hong Kong	No	Yes	1,148,403 (5.7)	822,405 (562,572)	21,461 (6,588)	86,527 (36,670)	218,010 (44,017)	35,388 (1,595)
China	No	No	1,075,574 (5.4)	249,192 (137,716)	361,993 (236,221)	464,389 (219,187)	0 (0)	4,362 (392)
Russia	N/D	N/D	676,944 (3.4)	263,458 (214,892)	2,720 (1,339)	198,074 (63,364)	212,692 (46,396)	14,600 (0)
EU	Yes	Yes*	623,908 (3.1)	238,386 (147,259)	135,298 (32,532)	23,183 (6,787)	227,041 (27,142)	68,552 (5,950)
Philippines	N/D	N/D	450,819 (2.2)	79,740 (75,791)	280,306 (83,739)	60,692 (29,369)	30,081 (6,790)	2,064 (659)
Taiwan	Yes	Yes	405,215 (2.0)	125,588 (104,941)	46,991 (18,363)	33,847 (14,583)	198,789 (35,354)	1,188 (282)
Australia	No	No	271,495 (1.4)	2,945 (644)	88,309 (32,564)	179,196 (56,059)	1,045 (178)	1,271 (183)
Netherlands**	Yes	Yes	235,935 (1.2)	38,465 (14,733)	54,138 (15,628)	8,692 (3,127)	134,640 (16,521)	11,390 (583)
Germany	Yes	Yes	57,751 (0.3)	24,939 (3,946)	6,662 (3,300)	4,642 (1,347)	21,508 (3,807)	21,098 (2,948)
Brazil	No	Yes	48,091 (0.2)	6,292 (69)	40,425 (22,356)	89 (11)	1,285 (273)	5,532 (18)
Ukraine	No	No	42,011 (0.2)	24,172 (11,745)	7,582 (2,344)	9,609 (3791)	648 (166)	11,604 (0)
Denmark	Yes	Yes	20,776 (0.1)	2,388 (487)	17,138 (3,736)	108 (24)	1,142 (187)	2,287 (445)
Sweden	Yes	Yes	7,768 (<0.1)	4,875 (1,138)	2,855 (716)	0 (0)	38 (6)	4,838 (1,137)

Data for this table were collected from Global Agriculture Trade Statistics in June 2012. Since data collection, some data may have been revised by USDA/FAS.

N/D=No Data.

* = with exceptions.

**= calculations include Netherlands Antilles.

^a^For egg exports, USDA/FAS measures quantities in two ways: 1) by metric ton (for eggs not sold in dozens) and 2) in dozens (for eggs sold in dozens). For consistency with other categories, we include only the quantity of egg exports reported by metric ton.

No human or animal subjects were used in this research. All persons consulted in the context of this study were serving in an official capacity as appointed or elected officials or as academic researchers; no personal information was sought.

## Results

### Antimicrobial use restricted

#### European Union

EU-wide, limitations on antimicrobial use began in 1997 when the EU banned using avoparcin for growth promotion [27]. In 1999, the bloc also banned growth promotion uses of tylosin, spiramycin, bacitracin, virginiamycin, carbadox, and olaquindox [27]. Remaining growth promotion uses were banned in 2006 [27]. Although the EU requires veterinary prescriptions to use antimicrobials in food animals, it allows member states to grant exemptions in certain cases. EU-wide information on the full economic impact of this ban is not yet available^a^, although some work has analyzed the specific economic impacts in several member states that are discussed later in this paper. The EU is an important export market for U.S. food animal products. Annually, the U.S. exports approximately $634 million of beef, pork, poultry, dairy, and eggs to the EU, or 3.1% of U.S. export sales across these food commodities (Table 2).

#### Taiwan

In 2005, Taiwan amended its Veterinary Drugs Control Act to ban AGPs and require veterinary prescriptions to use antimicrobials in food animals, according to the Taipei Economic and Cultural Representative Office in the U.S. Antimicrobials may be used in food animals to treat and prevent disease. Other information suggests that Taiwan has been trying to phase out antimicrobials for growth promotion but some antimicrobials are still permitted for feed use. These documents were not available in English for us to verify. Taiwan accounts for approximately 2% of annual U.S. export sales of food animal products, primarily in beef and poultry (Table 2).

#### Netherlands

In 1997, the Netherlands banned the antimicrobials olaquindox and carbadox; concerns had been raised about the carcinogenicity and genotoxicity of these drugs [28,29]. Two years later, the country began monitoring AMR in food and animal pathogens through its MARAN system [28]. Using MARAN data, it was reported that total sales of antibiotics licensed for therapeutic use in the Netherlands declined by nearly 32% (from 495 to 338 tonnes) between 2009 and 2011, surpassing a goal of a 20% reduction over that time period [30]. Veterinary prescriptions are reportedly required to use antimicrobials in food animals. We could not identify separate regulations pertaining to prophylactic antimicrobial use. Approximately 1.2% of annual U.S. export sales of food animal products are attributable to the Netherlands (Table 2).

#### Germany

Germany banned avoparcin in 1996. Furthermore, in addition to the 2006 EU-wide AGP ban, German law only allows antibiotics to be used for the treatment of diseased animals, not for growth promotion, and explicitly states that antibiotics cannot be used for diseases that arise as a result of “rearing conditions” [31]. Veterinary prescriptions are required to administer antimicrobials.

In 2008, Germany enacted its own national antibiotic resistance strategy, *Deutsche Antibiotika-Resistenzstrategie* or “DART”. That strategy includes AMR monitoring, improved data-sharing on resistance issues, and reducing antibiotic use through better environmental prevention of infectious diseases [32]. German state governments are responsible for assuring compliance [31].

In November 2011, Germany announced additional measures to control antimicrobial use in food animal production [31]. A central plank of this new effort is better monitoring of the quantities of antibiotics prescribed by veterinarians and the quantities actually consumed by food animals. Germany will also collect data on pharmaceutical use by the poultry industry for the first time. As a result of these provisions, the government expects to be able to track pharmaceutical use to individual animals [31]. Germany is expected to release data on overall quantities of pharmaceuticals used, including a geographic breakdown of veterinary antibiotic use. These new data will facilitate analyses of potential links between antibiotic use and resistance [31].

In February 2013, the German *Bundestag* (the lower house of the German parliament) passed a bill to require livestock producers to report regularly antibiotic use to German state governments [33]. The federal government would then use these reports to develop averages for antibiotic use. Producers that use antibiotics in excess of these averages would have their use policies reviewed by a veterinary authority, which would be empowered to direct the producer to adopt alternatives to antibiotic use. The measure must be passed by the *Bundesrat* (the upper house) before its enactment into law. Germany accounts for 0.3% of annual U.S. export sales of food animal products (Table 2).

#### Denmark

Denmark was an early adopter of antimicrobial control policies [27]. In 2000, it instituted a comprehensive national ban on AGP use, pre-dating the final EU-wide ban by six years [27]. The country also requires veterinary prescriptions to use antibiotics in food animals [27].

The first step toward Denmark’s ban on AGPs came in 1995 when Denmark banned avoparcin. In the same year, the Danish government placed a monetary cap on veterinarians’ profits from antibiotic sales. This removed an incentive for veterinarians to prescribe antimicrobials [27]. In 1998, the Danish poultry industry voluntarily stopped using AGPs [34]. Denmark’s swine producers also halted AGP use in finishing pigs before the national ban took effect [27,34]. From 1996–2003, the total use of antimicrobials in animals declined by 35%, although annual therapeutic use roughly doubled, due in large part to increased use in weaning pigs [35]. In 2010, the Danish swine industry adopted a voluntary ban on the use of third-generation cephalosporins [36].

Beyond these bans, Denmark has taken additional measures to limit antimicrobial use. In 2002, Denmark prohibited veterinarians from using fluoroquinolones except in cases where antimicrobial susceptibility testing of clinical isolates indicated that no other antibiotic could render effective treatment [27]. When fluoroquinolones are utilized, government officials must be notified. Three years later, in 2005, Denmark initiated biannual audits of the practices of swine veterinarians, eventually including all food-animal veterinarians [27]. In 2010, after observing an uptick in antibiotic use, the country initiated its “yellow card initiative,” which sets regulatory limits on antibiotic use based on the size of a swine farm, shifting the burden for minimizing antibiotic use from veterinarians to farmers [27,34]. Danish government officials report that since the initiative was implemented antibiotic use in food animal production has dropped by 25% [27].

In general, efforts to eliminate AGP use in poultry were implemented with few obstacles. The poultry industry had altered its production practices in the 1990s to reduce the occurrence of *Salmonella* infections in flocks; these changes had already resulted in lower antimicrobial use. Swine producers, however, faced difficulties in weaning piglets without antibiotics [27]. Danish producers reported increased treatment of piglets for diarrhea in pigs immediately following the ban, although diarrhea was typically not confirmed by veterinary or laboratory diagnosis [37]. Piglet mortality also increased but returned to previous levels after 1 year [38]. From 1992–1998, production of weaning pigs increased from 18.4 million pigs to 27.1 million pigs [34]. Ultimately, after the ban began, production did not drop off, and the upward trend in Danish swine production continued [14].

Compared to 1992, when antimicrobial use peaked, 2008 antimicrobial use in swine was down by half [14]. Poultry also did not experience long-term negative production impacts from the ban on AGPs, apart from a small increase in the feed-conversion ratio [39]. Danish industry representatives now report that changes to their production practices, such as later weaning, improved diet, and lower stocking densities, minimized negative impacts [27]. U.S. annual export sales to Denmark were $20 million in 2011, or about 0.1 percent of U.S. export sales of food animal products (Table 2).

#### Sweden

Sweden was the first country to ban AGPs in food animal production. In 1986, it instituted a national ban on AGPs and prohibited the use of antimicrobials absent a veterinary prescription [40,41]. Sweden also stipulated that antibiotics could only be used for “curing or preventing disease” [40]. The impact of this AGP ban was not uniform across animal species. The ban did not lead to negative clinical or economic repercussions in egg production since AGPs were not used in this area before the ban [40]. By 1986, AGPs were also rarely used in specialized beef production, so the ban did not result in negative production impacts in that sector either [40].

In turkeys, where antimicrobials sometimes used for growth promotion were reportedly only used to prevent necrotic enteritis, the ban did not result in negative clinical effects. Following the ban, broiler chicken producers were still able to use antimicrobials to prevent and treat infections. The industry chose to administer virginiamycin prophylactically to approximately 90% of broiler chickens in the country. In 1988, virginiamycin was replaced with penicillin, which was only administered in responses to outbreaks [40]. Reductions in broiler chicken production were not observed, and the industry remains strong; total production has nearly doubled in the 12 years following the ban [40]. Although there were post-ban increases in therapeutic applications of antimicrobials to help prevent or control outbreaks of necrotic enteritis for broiler chickens, those numbers have since dropped [40].

Pig producers did report seeing some initial negative results from the ban. Prior to 1986, regimens of olaquindox or mecadox were administered for growth promotion until 10–12 weeks and then avoparcin or virginiamycin regimens were administered to finishing pigs until slaughter at around 7 months [40]. While the health of finishing pigs and the cost of producing them remained unchanged, there were negative impacts among piglets. Piglet mortality increased in the year following the ban by 1.5 %, it took 5–6 days longer for pigs to reach 25 kilograms, and for the first four years following the ban, antibiotic use at therapeutic doses increased [40,41]. While government data indicate that antibiotic use dropped by approximately half by 1993, mortality and production time for piglets remained slightly elevated [41]. Compared to 1986–1987, 1997 average values for post-weaning mortality were 1–2% less and the age at 25 kg was 3.5-4.5 days less [40]. We were unable to obtain more recent information on these rates. In 2011, Sweden was responsible for less than 0.1 percent of U.S. export sales of food animal products (Table 2).

### Antimicrobial use semi-restricted

#### Mexico

In 2007, Mexico enacted its Federal Law of Animal Health, which restricts AGP use in animal feed and requires a veterinary prescription to use antimicrobials in food animals. The law banned most AGPs, but it provided exceptions for 15 drugs, including: avoparcin, vancomycin, bacitracin, tylosin, virginiamycin, avilamycin, bambermycin, spiramycin, salinomycin, and monensin. There are no current plans to phase out use of those drugs as growth promoters. Antimicrobial disease prevention and control regulations were unavailable in English. Mexico is the number one destination point for U.S. food animal product exports, responsible for 17.1 percent of U.S. export sales of those products annually (Table 2).

#### Japan

Japan does not have any restrictions on using antimicrobials for growth promotion, but its Food Safety Commission is reportedly considering restricting some uses of antibiotics. A veterinary prescription is required for antimicrobial use in food animals. Japan does not have any regulations pertaining specifically to antimicrobial use for the purposes of disease control and prevention. Japan is the second largest market for U.S. exports of food animal products, responsible for 15.2% of annual export sales of those products (Table 2).

#### South Korea

We could not verify whether South Korea currently restricts AGPs or requires a veterinary prescription to use antimicrobials. In July 2010, however, South Korea announced a ban on the “addition of antibiotics in animal feed…to strengthen the safety management of domestic livestock products” [22]. This ban, geared toward limiting the amount of drugs added into premixed animal feed, took effect in the second half of 2011. The ban is effectively a temporary hold on adding any antibiotics into commercial compound feed until a veterinary oversight system can be put into place, representing the final step in a multi-year phase-out of large-scale antibiotic additions without veterinary oversight [42]. Despite this targeted control effort, drugs can still be added to food, water, and injected in food animals on individual farms, but the addition of these drugs must occur at the farm location [42]. Once a veterinary oversight system is in place (reportedly expected no earlier than 2012, although we could not determine whether this has since occurred) antibiotics may be added into commercial compound feed at off-farm feed mills [42]. South Korea is responsible for 7.2% of annual U.S. export sales of food animal products and is a major market for U.S. exports of beef and pork (Table 2).

#### Hong Kong

Hong Kong does not have a comprehensive ban on AGPs but a representative of its Agriculture, Fisheries and Conservation Department states that Hong Kong producers do not utilize AGPs due to breed selection and the practice of bringing its animals to market at a later date. Select AGPs, including avoparcin, were banned in Hong Kong as of 2001. Veterinary prescription for antibiotic use in animals is also required by law. Hong Kong is responsible for 5.7% of annual U.S. export sales of food animal products (Table 2).

#### Russia

We were not able to verify Russia’s official policies on AGPs and veterinary prescription requirements. Media reports document instances where Russia has turned away meat imports because of its opposition to antimicrobial rinses used by the U.S. food animal industry [43]. Russia has regulations going into effect in July 2013 that may further limit antibiotic use though the impacts of these regulations remain unclear [44]. Russia is responsible for 3.4% of annual U.S. export sales of food animal products (Table 2).

### No antimicrobial restrictions

A number of countries do not have any current restrictions on AGPs and only have limited requirements to obtain veterinary prescriptions (Table 2). Canada, China (excluding Hong Kong), Australia, Brazil and Ukraine (listed in descending order of their financial contribution to U.S. export sales of food animal products) do not have any formal national restrictions on antimicrobial use for the purposes of growth promotion. Canada and Australia do report some limitations at the state or territory level.

Canada currently relies on voluntary actions to curtail use for growth promotion. Requirements to obtain veterinary prescriptions to use antimicrobials also vary by province, and the country does not have separate regulations for the use of antimicrobials for disease prevention and control.

China does not have any restrictions on AGPs and does not require veterinary prescriptions for antibiotic use. Detailed information about AGP use in China is difficult to obtain, but in 2007, the scientist that oversees China’s National Antibacterial Resistance Investigation Net (NARIN), estimated that almost half of the 210,000 tons of antibiotics produced in China are administered to animals via feed [45]. Brazil has not banned AGPs in livestock production and there is no requirement to obtain a veterinary prescription to use antibiotics in food animals, according to a representative from its embassy. Its regulations were not available in English. Ukraine has not banned AGP use, but its embassy reports that antibiotics are not used to produce food animals in Ukraine.

### Unknown

#### Philippines

We could not determine current policies in the Philippines related to restrictions on antimicrobial use in food animals or veterinarian prescription requirements. Efforts to communicate with Philippines officials were unsuccessful. The Philippines are responsible for 2.2% of annual U.S. food animal product export sales (Table 2).

## Discussion

We examined the international regulatory landscape related to the use of antimicrobials in food animal production. We sought to establish which jurisdictions have national requirements that veterinarians oversee antimicrobial use in food animals, and which jurisdictions have restricted the use of antimicrobials in food animals for growth promotion and, if information was available, disease prevention and control. We were able to ascertain answers to most of these questions for 17 political jurisdictions, and found that there are varying levels of antimicrobial controls in these settings, with restrictions on AGPs being the most common.

Six jurisdictions have stringent restrictions on antimicrobial use in food animal production. It is illegal to use antimicrobials for growth promotion in those settings and each requires a veterinary prescription to use antimicrobials in food animals. While the EU requires veterinary prescriptions for antimicrobial use, it exempts some of its 27 member states. Separately, five jurisdictions have fewer restrictions on antimicrobial use, with either full or partial AGP bans or veterinary prescription requirements. Five other jurisdictions do not have any restrictions on the use of antimicrobials for growth promotion at the national level or requirements to obtain veterinary prescriptions for their use (Table 2).

While a ban on growth promotion has been central to debates over antimicrobial use in the U.S., some jurisdictions’ policies have included more dynamic approaches as well. Denmark’s yellow card initiative, for example, combines robust surveillance of antimicrobial use with requirements to implement alternative measures when reported use is deemed excessive [27]. Proposed legislation in Germany would initiate a similar program [33]. These models recognize the importance of antimicrobials to animal health and the need for clinical judgments by qualified veterinarians, as well as the public health risks posed by misuse of these drugs. Denmark has also taken steps to limit financial conflicts of interest that may incentivize veterinarians to prescribe antibiotics excessively [27]. Neither approach has featured prominently in U.S. policy debates. A barrier to their adoption in the U.S. is the absence of a surveillance system for antimicrobial use in food animals.

Data on impacts of antimicrobial use restrictions on food animal production in these jurisdictions continue to be limited. Most information comes from the experiences of Denmark and Sweden, which were early adopters of antimicrobial controls. Their respective producers have suggested that stringent AGP controls can be implemented with minimal economic consequences for industry. Producers in Denmark and Sweden did not report increased mortality or encounter other obstacles in poultry, swine, beef and dairy products other than those described in this work. Producers did report elevated mortality and increased production time although those increases have been reduced over time as management practices have adapted [27,40,41].

Differences among jurisdictions with respect to food animal production policies have threatened U.S. access to export markets for these products. The EU has long prohibited the importation of U.S. beef produced with exogenous hormones, citing drug residues as a concern [26]. The bloc currently permits the importation of a limited quantity of beef derived from cattle certified by the U.S. government as “non-hormone treated” [26]. While the economic impact of this restriction is difficult to estimate, initial losses were projected at approximately $100 million per year [26]. Since 1997, the EU has also prohibited the importation of poultry processed with antimicrobial rinses, arguing that such treatments are less effective at reducing microbiological contamination than improved sanitation in production and processing [25]. Before 1997, U.S. poultry exports to the 15 countries that then comprised the EU were valued at $52 million; in 2011, poultry exports to those countries were valued at $13 million [25].

Beyond the EU, Taiwan had banned beef and pork products with detectable residues of ractopamine, a growth promotion and leanness drug used in the U.S. [46]. This ban was subsequently revised to permit ractopamine residues in beef up to 10 ppb while maintaining a zero-tolerance for ractopamine in pork [46]. U.S. access to the Russian market has been hampered in recent years by multiple issues, including antimicrobial drug residues and antimicrobial rinses [47]. The U.S. has long maintained that restrictions adopted by the EU, Taiwan, and Russia are not grounded in scientific evidence of human health risk, and that they instead represent economic protectionism [25,26,47].

Although international trade policies often hinge on myriad factors—for example, the most recent restrictions adopted by Russia appear to have been retaliation against criticism of the country’s human rights record by the U.S. Congress [48]—previous restrictions may still be instructive in the context of AMR. As concern over the contribution of food animal production to AMR in human pathogens increases, efforts to limit U.S. food animal exports further may emerge. In 2011, the EU adopted a resolution that called on its member states to “work towards an international ban on antimicrobials as growth promoters in animal feed, and to bring this matter up in its bilateral negotiations with third countries such as the United States” [22]. This suggests that, as with hormones and antimicrobial rinses, the EU may view antimicrobial use in food animals as a trade issue. Potential trade sanctions against the U.S. would likely lead to long-term consequences for the U.S. export market, stemming both from declining production capacities during sanctions as well as geographic dislocation of the market.

The World Trade Organization, which adjudicates trade disputes between its member states, relies principally on the opinions of the Codex Alimentarius Commission in cases involving food safety [49]. In 2005, the Commission published its “Code of Practice to Minimize and Contain Antimicrobial Resistance” [50]. The Code states, “The responsible use of veterinary antimicrobial drugs in food-producing animals…does not include the use for growth promotion of veterinary antimicrobial drugs that belong to or are able to cause cross resistance to classes of antimicrobial agents used (or submitted for approval) in humans in the absence of a risk analysis.” The Code also calls for veterinary oversight of antimicrobial use and emphasizes alternatives to antimicrobial use for disease prevention. In many cases, the current use of antimicrobials in U.S. food animal production appears to be inconsistent with these principles [16,51]. Although the FDA has recommended that pharmaceutical companies voluntarily withdraw approvals to market antimicrobials for growth promotion and require veterinary oversight of their use [4], the compliance of companies with these recommendations is uncertain. The agency has not moved to restrict antimicrobial use for disease prevention [4].

## Conclusions

This survey of national policies on antimicrobial use in food animal production demonstrates that multiple jurisdictions have adopted mandatory restrictions on antimicrobial use while the U.S. has slowly pursued a voluntary approach to this issue [4]. The experiences of Denmark and Sweden suggest that such restrictions may be adopted with minimal economic repercussions for food animal producers. Notably, the economic consequences of permitting antimicrobial use for growth promotion and disease prevention may be substantial given the importance of export markets to the U.S. food animal industry and the potential for trade barriers based on differences in antimicrobial use. To preserve the efficacy of antimicrobial drugs for treatment of infections in humans and other animals, the available scientific evidence indicates that the U.S. should restrict antimicrobial use [7,8,17]. The potential economic consequences of waiting to align antimicrobial use policies with those of current and potential export markets strengthens this case.

## Endnotes

^a^ The magazine *Bloomberg Businessweek* reported that the World Organization for Animal Health (better known by its French acronym, OIE) had estimated that the EU’s ban on antimicrobial growth promoters “reduced productivity for livestock breeders in the 27-nation bloc by about 5 percent compared with the rest of the world.” We were unable to locate a source for this report online, and multiple emails to the reporter and, in one case, the responsible editor went unanswered.

## Competing interests

The authors declare that they have no competing interests.

## Authors’ contributions

DFM contributed to study design, led data collection and analysis, and led the drafting of the manuscript. TJS originated the study, contributed to study design, participated in data analysis, and drafted sections of the manuscript. KEN contributed to study design, participated in data analysis, contributed to revisions, and provided expertise and oversight. All authors read and approved the final manuscript.
